# Observations on 3,590 Obstetric Cases

**Published:** 1915-10

**Authors:** 


**Observations on 3,590 Obstetric Cases.** Geo. G. Barnett, Ishpeming, Mich., Jour. of Mich. State Med. Soc., Sept., 1915.

The cases were observed in 30 years' practice, an average just short of ten per month. The birth-rate of Ishpeming has fallen from 40.2 per 1,000 population to 24.3 since 1897. The cases occurred in a total of 1846 women and resulted in the birth of 3,639 children. The youngest mother (primipara) was 14 years and 8 months; 3 others were 15; 12 at 16; 28 at 17; 50 at 18; 90 at 19; the maximum was reached at the age of 26 with 226 deliveries. 49 were delivered at the age of 42, 26 at 43; 19 at 44; 11 at 45; 3 at 46; 4 at 47; none beyond this age. The oldest primipara was 40. According to number of labors, there were 670 cases of primiparturition (18.6 per cent); the successive numbers being: 615, 640, 540, 442, 334, 271, 208, 155, 11, 85 (tenth labor), 63, 38, 25, 17, 10 (fifteenth labor), 4, 1, 1, (eighteenth labor).

The presentations were as follows: vertex 3,415 (slightly over 95.5 per cent); breech 99, foot, 46, face 32, transverse 21, arm and shoulder 18, breech and hand 5, both hands 1, shoulder 2—total 3,639.

Weight of infants: 6 weighed 2 pounds; 17 at 3 lbs.; 43 at 4 lbs.; 91 at 5 lbs.; 263 at 6 lbs.; 799 at 7 lbs.; 869 at 8 lbs.; 684 at 9 lbs.; 257 at 10 lbs.; 54 at 11 lbs.; 22 at 12 lbs.; 5 at 13 lbs.; 1 at 15 lbs.; and 1 at 16 lbs. The average weight of 3,112 infants was 7.87 lbs. no account having been taken of fractions.

The birth incidence by months did not vary much—the minimum being 243 for November, the maximum 339 for August.

The births between 1 a. m. and noon numbered 1,984, those between 1 p. m. and midnight 1,595. The minimum was 109 at 3 p. m., the maximum 206 at 1 a. m.

Still births numbered 113 (3.1 per cent.) males 68, females 45.

Placenta praevia occurred in 11 cases with 2 deaths.

There were only 28 illegitimate births (0.8 per cent), male 15, female 13. The general average for the state, 1906 to 1913 was 1.7 per cent illegitimate, and 3.5 per cent. still born.

Version was performed 60 times with 33 living children. There were 60 instances of two deliveries in a year, 26 at full term, 34 premature Triplets were encountered once. Eclampsia occurred in 9 cases with 1 death, well within Hirst's estimate of an incidence of 1 per 300 of a mortality of 30 per cent. 19 maternal deaths occurred in the whole series, a trifle over 1-2 per cent. Two cases had pneumonia, 1 pneumonia with mania, 1 typhoid, 1 appendicitis, 1 rheumatic fever, 1 pernicious anaemia, a total of 5 from extrinsic causes. The causes of death due to labor or its direct complications were placenta praevia 2, eclampsia 1, shock and haemorrhage 1, sepsis and peritonitis 10. Thus it will be seen that the actual death rate due to pregnancy was less than 1-2 per cent.

Abnormalities noted: Spina bifida 5, imperforate anus 2, acephalous monsters 3, defective digits, etc. 4, supernumerary digits 2, webbed digits 1. 2 cases of hydatiform mole were seen.

The average length of umbilical cord was 22 inches but lengths of 29, 31, 37 and 38 inches were observed. There was one case of prompt and painless labor in a 3-para named Quick.

The longest interval between marriage and first child was 13 years, the shortest 10 days except one in which the interval was minus 3 days. A grandmother was noted at the age of 32. (The editor had this experience in general practice, years ago, and also had a family in which females were living from great great grand-daughter to great great grandmother, the middle one dying first at about 40.) An example of prolificity was the following: children born April 4, 1900, March 30, 1901, July 16, 1902, July 10, 1903, June 28, 1904, August 23, 1905, and six more up to December 11, 1914.

(Note: This article is not only of the highest value in itself but is one of the best examples of careful recording of private practice that we have encountered. It is obvious that such records, for any kind of medical case, in the class of patients seen in private practice are much more representative than experience derived from hospital experience. Moreover, accuracy is much more likely to be attained in personal records than in those compiled from a variety of attendants. We call special attention to the desirability of statistic articles of this nature and hope our readers will submit such for publication in the **Buffalo Medical Journal.**

